# Clinical and Molecular Aspects of Iron Metabolism in Failing Myocytes

**DOI:** 10.3390/life12081203

**Published:** 2022-08-08

**Authors:** Bogna Kozłowska, Barbara Sochanowicz, Leszek Kraj, Małgorzata Palusińska, Piotr Kołsut, Łukasz Szymański, Sławomir Lewicki, Marcin Kruszewski, Marta Załęska-Kocięcka, Przemysław Leszek

**Affiliations:** 1 Department of Heart Failure and Transplantology, The Cardinal Stefan Wyszyński National Institute of Cardiology, Alpejska 42, 04-628 Warsaw, Poland; 2 Center of Radiobiology and Biological Dosimetry, Institute of Nuclear Chemistry and Technology, Dorodna 16, 03-195 Warsaw, Poland; 3 Department of Oncology, Medical University of Warsaw, 1A Banacha Str., 02-097 Warsaw, Poland; 4 Department of Molecular Biology, Institute of Genetics and Animal Biotechnology, Polish Academy of Science, Postępu 36A, 05-552 Magdalenka, Poland; 5 Department of Cardiac Surgery and Transplantology, The Cardinal Stefan Wyszyński National Institute of Cardiology, Alpejska 42, 04-628 Warsaw, Poland; 6 Faculty of Medical Sciences and Health Sciences, Kazimierz Pulaski University of Technology and Humanities, 26-600 Radom, Poland; 7 Department of Molecular Biology and Translational Research, Institute of Rural Health, Jaczewskiego 2, 20-090 Lublin, Poland

**Keywords:** heart failure, myocardial iron metabolism, oxidative stress, myocardial gathering proteins expression, human model

## Abstract

Heart failure (HF) is a common disease that causes significant limitations on the organism’s capacity and, in extreme cases, leads to death. Clinically, iron deficiency (ID) plays an essential role in heart failure by deteriorating the patient’s condition and is a prognostic marker indicating poor clinical outcomes. Therefore, in HF patients, supplementation of iron is recommended. However, iron treatment may cause adverse effects by increasing iron-related apoptosis and the production of oxygen radicals, which may cause additional heart damage. Furthermore, many knowledge gaps exist regarding the complex interplay between iron deficiency and heart failure. Here, we describe the current, comprehensive knowledge about the role of the proteins involved in iron metabolism. We will focus on the molecular and clinical aspects of iron deficiency in HF. We believe that summarizing the new advances in the translational and clinical research regarding iron deficiency in heart failure should broaden clinicians’ awareness of this comorbidity.

## 1. Introduction

Iron is a crucial micronutrient necessary for many biological processes such as oxygen storage and transportation (as a component of hemoglobin, myoglobin, oxidative enzymes, and respiratory chain proteins) and the synthesis and degradation of proteins, lipids, or nucleic acids (Fe-S cluster formation). Therefore, iron homeostasis is crucial for proper cell function and depends on the absorption and transportation of iron into the cell [1,2].

The human body contains 3.5 to 4.5 g of iron, depending on sex. The majority of iron is bonded to in red blood cells (60%). Approximately 25% of iron is stored in ferritin (FT) and hemosiderin in the liver, spleen, and bone marrow. Another 5%–6% is associated with myoglobin in muscles. Less than 1% is bound to transferrin (Tf). Iron appears in two forms in the human body, including ferric iron (Fe3^+^) (the oxidized form with limited solubility) and ferrous iron (Fe2^+^) (absorbable iron) [1,3].

Iron homeostasis is mainly maintained and regulated by iron absorption in the duodenum and the proximal jejunum. Depending on the circumstances, iron absorption in the duodenum can increase from 1–2 mg in normal conditions to two–three times that in the case of an iron deficiency. Around 20% of Fe2^+^ and 5% of Fe3^+^ is absorbed from food. In the enterocytes, iron is stored or exported from the cell into the circulating blood through ferroportin [FPN]. Enterocytes are replaced within three days, therefore, lots of iron is lost via the stool [4]. Fe2^+^ release into the blood depends on the hephaestin (Heph) protein that oxidizes Fe2^+^ to Fe3^+^ to allow iron binding to apotransferrin (ApoTf). After binding two iron atoms, the protein is called holoTf and delivers iron to the heart and all other tissues and organs. holoTf can be captured by transferrin receptor-TfR, the receptor-transferrin complex is absorbed into the cell by endocytosis, and iron is released into the cytoplasm. Inside the cell, iron ions are stored in the form of FT and hemosiderin [5], and in the form of the labile iron pool (LIP). LIP may be highly toxic to the cells [6]. Thus, proper iron homeostasis is crucial for cell function, especially in high-energy-demand cells such as skeletal muscles and cardiomyocytes.

Heart failure (HF) is a disease associated with cardiac remodeling, involving many factors, including functional, metabolic myocyte remodeling, fibrosis, and inflammation leading to attenuated heart functionality. An adequate level of iron in the heart plays a vital role in the proper functioning of this organ. Among many other factors, both iron deficiency and iron overload may develop heart damage. What is interesting is that in heart failure, iron deficiency (ID) exists in 35%–55% of patients (with a reduced ejection fraction—HFrEF) [6], 59% of patients (with preserved ejection fraction) [7], and 80% of patients (with acute HFrEF) [4]. Therefore, an intravenous iron replenishment is becoming a more frequent form of treatment, which in some cases may lead to iron overload. However, iron overload leads to oxidative myocardial damage, as the iron excess exerts a harmful effect mainly related to the production of intracellular reactive oxygen species [8,9]. Here, we describe the current, comprehensive knowledge about the proteins involved in iron metabolism in cardiomyocytes, particularly considering its role in failing hearts.

## 2. Iron Homeostasis in the Heart

**Figure 1:**
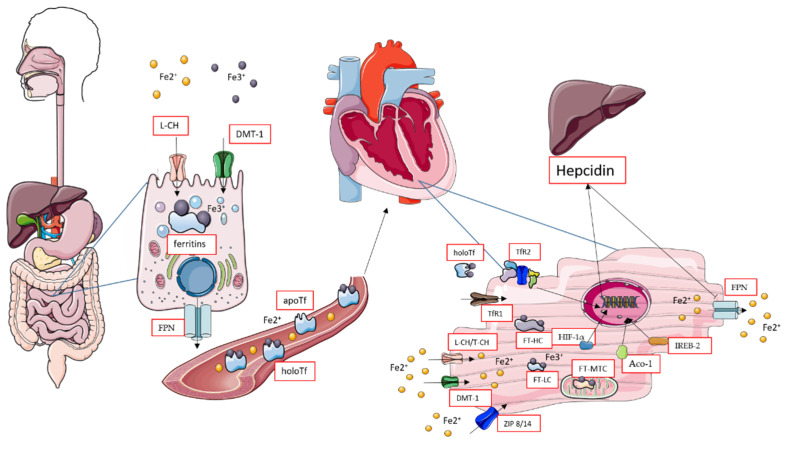
Absorption of iron by the intestines and turnover in the heart. Aberrations: Aco-1—aconitise (aso IREB-1); DMT-1—divalent metal transporter 1; Fe2^+^,Fe3^+^—iron ions on 2^+^, and 3^+^, oxidation state; FPN—ferroportin; FT—ferritins: HC (heavy chain), LC (light chain), MTC (mitochondrial chain); HIF-1α—hypoxia-inducible factor α; IREB-2—iron-responsive binding element-2; L-CH—calcium channels L type; Tf—transferrin: apo—without iron ions, holo—with bounded iron; TfR1/TfR2—transferrin receptors 1 or 2; ZIP 8/14—Zrt-, Irt-like proteins 8 or 14. The figure was created using SMART (Servier Medical ART)-modified graphics, licensed under a Creative Commons Attribution 3.0 generic license.

Several proteins are involved in iron transport, storage, and regulation to maintain cardiac iron homeostasis. Generally, the iron-gathering proteins may be divided into iron transport, storage, and regulation. Iron transport from the digestive tract to the heart is shown in Figure 1.

### 2.1. Iron Transport

Iron enters the cardiomyocytes through several proteins: transferrin receptor 1/2-TfR1/TfR2, divalent metal transporters-DMT1, non-specific ion channels (L-type calcium channel—L-CH), and ZIP channels.

#### 2.1.1. Transferrin Receptor 1

The leading iron transporter into the cardiomyocyte is TfR1. TfR1 is a transmembrane glycoprotein composed of two monomers linked by disulfide bonds. After one holotransferin is bound to each monomer, the complex (holoTf-TfR1) is transported to the cell by clathrin-dependent endocytosis. This process begins by formation on the cells’ surface clathrin-coated pits (CCPs) and requires the adaptor protein AP2 [10]. Then, iron from the endocytes is released by a decrease in pH, and both proteins are transported back to the cell surface in endosomes—Tf is released to the bloodstream (Apo-Tf), and TfR1 is located on the cell membrane and ready for another iron holo-Tf transport.

Several mechanisms regulate TfR1 expression. Generally, the expression of TfR in the cardiomyocytes is regulated by iron status. A decrease in the myocardial’s iron concentration activates iron regulatory proteins (IRP1 and IRP2), which control the post-transcriptional expression of proteins responsible for iron turnover in the cell. IRPs bind to IREs in the mRNA 3′-untranslated region (UTR) to stabilize the promoter of TfR gene (*TFRC*) mRNA expression [11]. A low concentration of iron activates IRPs, which causes an increase in mRNA expression of TfR1 and a decrease in ferritin expression. This causes an increase in the cellular iron concentration, inhibiting IRPs function and reversing the process. The *TFR1* knockout in the heart of mice causes iron deficiency and, ultimately, fatal heart failure and death in the second week of life, mainly due to the dysfunction of mitochondrial respiration and mitophagy [12]. TfR1 is expressed in various types of cells and plays a unique role in adipocytes. mRNA silencing of *TFR1* in thermogenic adipocytes causes a reduction in thermogenic gene programs and mitochondrial integrity [13]. Mice lacking *TFR1*, especially in adipocytes, have iron deficiency, mitochondrial dysfunction, impaired thermogenesis, increased insulin resistance, and low-grade inflammation [14]. In thyroid cancer cell lines, downregulation of TfR1 inhibited the ERK pathway and reduced c-Myc expression, which contributed to increased apoptosis [15]. Another modulation mechanism of TfR1 expression is connected with hypoxia-inducible factors (HIF-1α and HIF-2α). The hypoxia-response element (HRE) is localized in the promoter of the TfR gene. In hypoxia or iron deficiency, the expression of hypoxia-inducible factors (HIF-1α and HIF-2α) increases, and these proteins bind to the HRE in the promoter of *TFRC*, thereby promoting *TFRC* transcription [16]. Besides protein interactions, some researchers described the role of long non-coding RNA in regulating TfR-1 activation, which caused ferroptosis of cardiac cells and cardiac hypertrophy [17].

#### 2.1.2. Transferrin Receptor 2

Another receptor in cardiomyocytes’ iron turnover is a glycoprotein—transferrin receptor 2 (TfR2). In general, TfR2 appears in two forms, the full-length TfR2α and the shorter-form TfR2β, for which expression is regulated by different specific promoters [18]. Contrary to TfR1, regulation of these receptors is devoid of IRE and is regulated mainly by transcription factors such as enhancer-binding protein-α; GATA-binding factor 1, and hepatocyte nuclear factor 4α [19]. TfR2α is responsible for the regulation of liver hepcidin. The function of TfR2β is generally unknown, however, some studies have suggested that this isoform may be involved in the regulation of iron homeostasis in the heart, where very high TfR2β levels were observed [20,21]. Impaired function of or mutations in the *TFR2* gene lead to significantly decreased hepcidin expression, resulting in increased iron uptake from the digestive tract and hemochromatosis [18].

#### 2.1.3. Divalent Metal Transporter 1

Divalent metal transporter 1 (DMT1) is another important transporter protein involved in the uptake of non-transferrin-bound iron (NTBI) by cardiomyocyte cells. DMT1 expression is strongly upregulated by iron deficiency in the rat heart [22] and down-regulated in the mice heart under iron overload conditions [23]. DMT1 appears in two forms—containing IRE and devoid IRE, as shown on animal models [24]. The presence of an IRE-like motif suggests that IRPs, similar to TfR receptors, could regulate DMT1. However, rat studies have shown that iron status has no significant effect on the mRNA expression level of *DMT1* (both IRE and non-IRE) in the heart [24]. The main factor regulating *DMT1* transcription is HIF2α [16]. Studies performed in mice revealed that a chronic iron deficiency triggers an increase in the expression of the *DMT1-IRE* mRNA isoform in wild-type mice, which was stimulated by HIF-2 α [25]. Moreover, in the mice model of targeted disruption of the *DMT1 3′IRE* (DMT1^IREΔ^), the authors showed that this domain is required for proper post-transcriptional stimulation of DMT1 expression, and the disruption decreased the level of iron in the serum during early life [26]. DMT-1 overexpressed in transgenic mice resulted in selective iron accumulation in the brain, mainly in the substantia nigra [27].

#### 2.1.4. Zinc Transporters

Proteins from the ZIP family (Zrt-, Irt-like proteins), the main Zn transporters, also transport NTBI to the cell’s cytoplasm. Research indicates that ZIPs may play an important role in cardiomyocytes’ iron homeostasis. One of them is ZIP8. Iron-overload conditions present in the mouse model of β-thalassemia upregulate the expression of the ZIP8 in heart tissue compared to wild-type mice [23]. Moreover, ZIP8 is regulated by cellular iron loading but at a post-transcriptional level. Iron loading increases ZIP8 protein levels, whereas ZIP8 mRNA did not vary [28]. The role of ZIP8 is not fully understood but is essential for embryonic development, as shown in studies in knockout mice [29,30]. Loss of ZIP8 expression below 10%–15% compared to wild-type mice is lethal to their embryos [30]. Another member of the ZIP family involved in iron uptake is ZIP14. Experiments on ZIP14 knockout mice showed reduced NTBI uptake by the spleen and liver, however, there were no significant changes in heart Fe concentration [31]. The expression of ZIP14 in the human heart is significantly higher than the level of ZIP8 [28], however, unlike ZIP8, it is not regulated by iron status [22]. Additionally, the expression profile of both genes differs significantly, suggesting their various roles [28].

#### 2.1.5. Calcium Channels

Other vital routes of iron influx into cardiomyocytes are cardiac calcium channels, including L-type (L-CH) and T-type (T-CH). Both types of channels mediate the import of iron not bound to transferrin. Cardiac calcium channel influx is believed to be the primary mechanism for non-transferrin-bound iron uptake. The channels are highly expressed in cardiomyocytes and primarily transport Ca^2^⁺ and Fe^2^⁺. Depolarization of the membrane causes an opening of the channel, thus allowing the influx of calcium (Ca^2^⁺) ions into the cell. [32]. However, when the concentration of non-transferrin Fe^2^⁺ in the blood increases, the iron is transported to the cells. This may lead to excessive iron overload and iron toxicity, as was shown in various experimental models. Iron overload in wild-type mice was associated with increased mortality, cardiomyocyte dysfunction, myocardial fibrosis, and elevated oxidative stress. Overexpression of L-CH channels in transgenic mice almost doubled the oxidative stress levels compared to the wild type. Both mice treated with L-CH blockers (amlodipine and verapamil) had significantly lowered myocardial iron accumulation and levels of oxidative stress markers [33]. Moroever, the dual T-type and L-type calcium channel blocker, efonidipine, caused a significant reduction in iron uptake in cardiomyocytes isolated from genetically altered β-thalassemic mice [23], regulating iron levels as well as improving mitochondrial and cardiac function in iron-overload β-thalassemic mice and wild-type mice [34]. Iron uptake also affects levels of L- and T-type calcium channels. In mice, iron overload significantly increased both L- and T-type calcium mRNA and protein expression in the heart [35]. Moreover, iron overload causes iron-induced changes in calcium signaling and disturbs the metabolism and function of human-induced pluripotent stem cells derived from cardiomyocytes [36] and astrocytes [37]. On the other hand, in animal models, calcium chanel blockers have shown a reduction in cellular iron uptake [34], which is not confirmed in human models [38].

#### 2.1.6. Ferroportin

The protein responsible for iron excretion from the cardiomyocytes is ferroportin (FPN). It is an iron export protein that transports Fe2^+^ into the blood (from cells to circulation). FPN is the only known protein responsible for iron extraction for the cells in mammals; therefore, its mutation generally causes hemochromatosis [39]. Important for iron extraction are mutations not only in iron but also in the hepcidin binding site. A point mutation in murine *FPN* locus (C326S) caused fatal iron overload, with increased plasma iron and ferritin levels, transferrin saturation, and hepatic iron overload [40]. However, there are also mutations in the *FPN* gene that disrupt hepcidin-induced degradation of FPN, which, in turn, causes the excessive iron-export activity of the protein and leads to iron deficiency. Zhang et al. observed in erythroblast-specific *FPN* knockout mice an iron deficiency in blood with not-affected hepcidin levels. [41]. Red blood knockout cells were also more prone to hemolysis induced by oxidative stress.

### 2.2. Iron Storage

The other major group that plays a vital role in iron homeostasis in human hearts are ferritins—proteins responsible for storage, sequestration, and detoxification of iron excess. Cytosolic ferritin consists of 24 subunits complex of two types, ferritin heavy chain (FT-HC) and light chain (FT-LC), and also mitochondrial ferritin (FT-MTC). Ferritin stores iron in a soluble, nontoxic, and readily available form; its enzymatic activity allows for the conversion of Fe2^+^ to Fe3^+^ and the packing of iron into the mineral core. As only FT-HC has this ferroxidase activity, it plays an important role in the detoxification of toxic iron and many others. The expression of the ferritin is IRE-dependent. Inactivation of the *FT-HC* gene by homologous recombination in mice causes the death of embryos at an early stage of development. [42]. Heterozygous of *FT-HC* (±) have 7–10-fold elevated serum FT-LC levels, without any changes in serum iron concentration, and higher FT-LC levels in the heart [43]. Additionally, following iron injections, stimulation of FT-HC production rose two-fold in the heart and was identical in all the organs. FT-HC deletion in mice significantly facilitates cardiomyopathy by activation of ferroptosis [44]. It also increased the labile iron pool and enhanced reactive oxygen species formation and mitochondrial depolarization [45]. Overexpression of FT-HC generally provides for iron depletion and dysregulation of the iron metabolism in cells [46].

The FT-LC subunit takes part in iron-core formation inside the ferritin, where iron is stored. The proportion of FT-HC to FT-LC varies and depends on tissue type, physiologic status, and inflammation [47]. Mutation of *FT-LC* gene in mice leads to develop of neurodegenerative disease, accumulation of ubiquitinated proteins, formation of ferritin inclusion bodies, and iron overload [48,49]. Overexpression of the FT-LC in *Drosophila melanogaster* did not significantly change the health parameters in young flies, however, it caused iron deposition in the optic lobes and late-onset behavioral defects in older flies [50]. Moreover, overexpression of FT-LC protein is noticed in various tumors, which plays an important role in the proliferation, survival, and progression of tumor growth [51].

The last of the ferritins in cells is mitochondrial ferritin (FT-MTC). It is an IRE-independent gene encoded on chromosome 5q23.1, with high homology to *FT-HC*. The protein is expressed mainly in cells with high metabolic activity such as cardiomyocytes, neurons, or spermatocytes [52]. The role of this protein in iron metabolism is still questionable due to both mutations [53] and overexpression [54] of *FT-MTC* gene did not affect iron metabolism. The role of this protein in the heart may bring the studies on Friedreich ataxia closer, in which the FT-MTC gene is upregulated, and cardiomyopathy is the highest cause of death [55]. Some authors postulated that overexpression of FT-MTC protein may protect mitochondria in cardiomyocytes from ferroptosis induced by mitochondrial LIP and oxidative stress [52].

### 2.3. Iron Regulation

The most crucial iron regulator in the body is hepcidin (HEPC)—an iron-regulatory hormone encoded by HAMP (hepcidin anti-microbial peptide; type 2B) and mainly produced by the liver [56]. Hepcidin production is induced by iron loading and inflammation. An increase in hepcidin concentration inhibits iron loading. The regulation of HEPC expression in response to an increase in iron concentration is controlled by two pathways. First is the bone morphogenetic protein (BMP)–mother against the decapentaplegic (SMAD) pathway. In iron excess, BMP (produced by the liver) interacts with a dimeric serine-threonine kinase receptor (BMPRII), activating the dimeric BMP type I receptor (BMPRI). Catalytically active BMPRIs phosphorylate SMAD1/5/8, upon SMAD4 binding, translocates to the nucleus to modulate the expression of Hepc [57,58]. The second pathway is the hemochromatosis type 1 protein (Hfe)/transferrin receptor 2 (Tfr2) complex involved in the Hepc response to iron growth by activation of the Erk1/2 and MAPK cascade. Studies in mice confirm the importance of this pathway. Mice with the *HFE* -/- or *TFR2* mutation show a limited ability to increase hepcidin with a strong iron overload [59]. In addition, *HFE* and *TFR2* knockout mice showed lower pErk1/2 and pSmad1/5/8 proteins, which means that Hfe and Tfr2 proteins participate in the regulation of both described pathways [60,61].

Iron circulation in the body is regulated at many levels, including cellular. As described above, cells respond to intra- and extracellular iron concentrations by modulating the expression of specific proteins responsible for transport, storage, and iron excretion. This modulation is controlled mainly by IRP-1/IRP-2, hemojvelin (HJV), and the hypoxia-inducible factor (HIF-1α).

The IRE/IRP system involves the iron regulatory proteins 1 (IRP1) and 2 (IRP2), which are cytoplasmic polypeptides belonging to the iron–sulfur family isomerases. IRP1 consists of 889 and IRP2 of 964 amino acids [62]. IRPs bind to the iron-responsive element (IRE) located in mRNA’s untranslated region (UTR). IREs are phylogenetically conserved hairpin structure motifs of 26–30 nucleotides [63]. IRPs can bind to IREs located at the 5′UTR inhibiting mRNA translation or at the 3′UTR, protecting the mRNA from degradation. On the post-transcriptional level, IRE/IRP is involved in the transcription of various genes (Table 1), but the most important are TfR and ferritin. Iron-concentration changes in the cells activate IRP1 and IRP2, which bind to IREs in the 3′UTR mRNA of TfR to stabilize the promoter of the TfR gene [11]. On the other hand, low iron concentrations activate IRPs, which causes an increase in mRNA expression of TfR1 and a decrease in ferritin expression. The role of the IRE/IRP system in regulating heart iron homeostasis was confirmed in a mouse model and a human heart. Deletion of Irp1 and Irp2 in murine cardiomyocytes results in an impaired left ventricular response to dobutamine and aggravation of myocardial infarction, whereas IRE-binding activity is reduced in the failing human heart [64].

HIF family members, hypoxia-inducible factors 1 and 2 (HIF-1 and HIF-2), exist as heterodimers with a ubiquitous b subunit (HIF-1beta) and an oxygen-sensitive a subunit (HIF-1 α or HIF-2 α). The family of HIF transcription factors controls key physiological processes, including erythropoiesis, energy management and metabolism, angiogenesis, cell proliferation, and cell migration. In hypoxic conditions, there is no HIF degradation, which results in increased EPO production by both the kidneys and the liver. The synthesis of EPO is mediated mainly by HIF-2α. In iron deficiency, absorption is increased by reducing the level of hepcidin, which promotes iron export from enterocytes, additionally stabilizing HIF-2α [66]. IRP1 binding to HIF-2α 5′IRE represses the latter’s translation and decreases erythropoietin production, limiting erythropoiesis and iron consumption [67]. This process was shown in *IRP1*-knockout mice, where young, iron-deficient animals have transient polycythemia that recurs in adult mice with normal iron levels. [68,69]. Moreover, HIF-2α activation is modulated by intracellular iron through regulation of prolyl hydroxylase (PHD) activity, which requires iron as a cofactor. Prolyl hydroxylase, the enzyme that induces degradation of HIF-2α, is iron-dependent and is, thus, inactive in iron deficiency.

**Figure 2:**
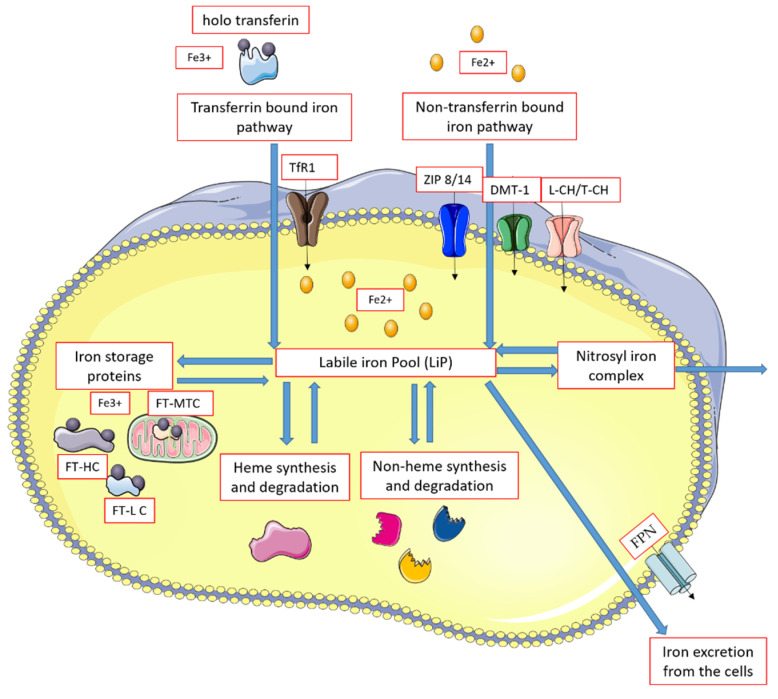
Intracellular iron turnover. Aberrations: DMT-1—divalent metal transporter 1; Fe2^+^,Fe3^+^—iron ions on 2^+^, and 3^+^, oxidation state; FPN—ferroportin; FT—ferritins: HC (heavy chain), LC (light chain), MTC (mitochondrial chain); HIF-1α—hypoxia-inducible factor α; HJV—hemojvelin; L-CH—calcium channels L type; Tf—transferrin: apo—without iron ions, holo—with bounded iron; TfR1—transferrin receptors 1; ZIP 8/14—Zrt-, Irt-like proteins 8 or 14. The figure was created using SMART (Servier Medical ART)-modified graphics, licensed under Creative Commons Attribution 3.0 generic license.

Cellular turnover (transportation, storage, use, and elimination) of iron ions is shown in Figure 2.

## 3. Dysregulation of Iron Metabolism in the Failing Heart

**Figure 3:**
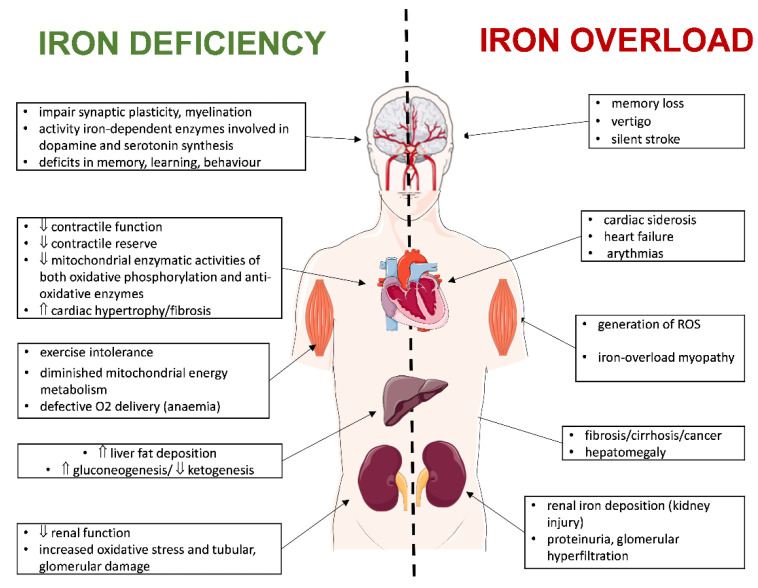
Effect of iron overload and deficiency in the organism. The figure was created using SMART (Servier Medical ART)-modified graphics, licensed under a Creative Commons Attribution 3.0 generic license. ↑—increase, ↓—decrease.

Proper iron distribution in the heart is crucial for its proper function. Both an overload and iron deficiency contribute to myocardial damage and heart failure (Figure 3). Below, we describe this in detail.

### 3.1. Iron Overload

Iron overload can be divided into two categories: primary iron overload, which emerges from hereditary genetic mutations, and secondary iron overload, caused by excessive iron intake.

Primary iron overload is caused by hereditary hemochromatosis, caused by mutations in the five genes coding the various iron-transporting and iron-sensing proteins. Interestingly, these mutations are the most common genetic disorders in the European population [70]. There are three main mechanisms responsible for hemochromatosis:

Loss-of-function mutations in the genes coding for the proteins (human hemochromatosis protein—HFE, hemojuvelin—HJV, transferrin receptor 2—TfR2) of iron-sensing complex disable the production of hepcidin in response to a rising iron load (Types 1, 2A, and 3, respectively);

Loss-of-function mutations in the gene coding for hepcidin (Hamp) dysregulate the production of hepcidin (Type 2B);

Gain-of-function mutations of the gene coding ferroportin (Slc40a1), which loses the ability to interact with hepcidin (Type 4B).

The three main mechanisms of mutations leading to hemochromatosis are associated with either the synthesis or regulation of hepcidin, with the main mutation in the HFE gene leading directly to the dysregulation in hepcidin synthesis. Low hepcidin levels, through the attenuated effect on macrophages and enterocytes, lead to increased iron release from macrophages and increased intestinal iron uptake. Therefore, people with HFE-gene mutations cannot reduce their iron absorption in response to elevated iron levels in the body. In addition, blood transfusion is a factor that can induce diastolic and systolic LV dysfunction in secondary iron overload (Table 2) [71].

#### 3.1.1. Labile Iron Pool

Through concerted action of iron delivery and iron-storage, proteins assure efficient iron chelation that prevents its availability of iron as a catalyst of the Fenton reaction, so continuous demand for iron ions for the synthesis of a plethora of iron-containing proteins (e.g., Fe-S clusters proteins, heme proteins, and other proteins containing iron ions at their active centers) results in a trace amount of iron ions that are bound neither to iron-storage proteins nor iron-transport proteins. This small amount of “free” iron ions is called the “labile iron pool” (LIP) or “chelatable iron pool”. LIP is defined as a low-molecular-mass pool of weakly chelated iron ions that pass through the cell. It likely consists of Fe2^+^ and Fe3^+^ complexed by various low-molecular mass ligands with a low affinity for iron ions such as citrate, phosphate, carbohydrates, carboxylates, nucleotides, nucleosides, polypeptides, and phospholipids [8].

An important player in LIP turnover seems to be histidynyl- and dinitrosyl iron complexes (DNICs), consisting of low or high molecular mass ligands with tiol or histidine groups, nitric(II) oxide, and iron ions. In physiological conditions, DNICs are formed in events of intensified NO production, such as inflammation, ischemia/reperfusion, and neurodegeneration. DNICs have been proposed to be NO donors in many NO-dependent processes, however, their role in iron chelation has also been considered [9].

In general, LIP is considered as a pool of iron ions readily available for the Fenton reaction and likely responsible for the detrimental effects of iron in living organisms.

#### 3.1.2. Iron-Overload Consequences

Though iron is indispensable for all living organisms, its excess might have detrimental effects. Iron ions may catalyze the Fenton reaction, resulting in hydroxyl radical formation, one of the most reactive chemical moieties that can oxidize almost every molecule. In iron overload, a fraction of free iron ions in the blood are elevated, which facilitates the influx of free Fe2^+^ ions into cells and tissues. This free iron ion influx to the cells is excitable through voltage-dependent Ca2^+^ channels [72] and/or divalent metal ions transporters, such as ZIP8, ZIP14, and DMT1 [28,73,74]. Cardiomyocytes contain both voltage-dependent channels and divalent metal ions transporters, and, therefore, iron overload, such as hemochromatosis, results in severe, iron-dependent damage to the cardiac tissue. The primary mechanism of iron-overload damage is an excessive generation of hydroxyl radicals and other reactive oxygen species (ROS), accompanied by an imbalance in ROS degradation, known as oxidative stress. Once the production of ROS surpasses the capacity of intracellular antioxidant systems, the accumulation of ROS damages the cellular components, such as nucleic acids, proteins, and lipids. In cardiomyocytes, oxidative stress manifests as (i) impaired excitation/contraction coupling, (ii) elevated cytoplasmic level of Ca2^+^ ions due to impaired function of SERCA, (iii) impaired oxidative phosphorylation and ATP deficiency due to the mitochondrial membranes lipid peroxidation, and (iv) damaged DNA and mitochondrial DNA [75]. Iron overload at the tissue level promotes the proliferation of fibroblasts and their differentiation to myofibroblasts, leading to fibrosis of the cardiac tissue and/or alterations of the macrophage–cardiomyocyte interaction, resulting in inflammation and subsequent cardiac-tissue remodeling. Both can evoke diastolic dysfunction, arrhythmias, and restrictive cardiomyopathy [76].

Overwhelming lipid peroxidation due to the excess of intracellular redox active iron ions is characteristic of iron overload. Lipid peroxidation disturbs cell membrane fluidity and functionality, leading to the activation of controlled cell death, known as ferroptosis. Ferroptosis is not yet a fully understood mechanism of cell death, characterized by mitochondrial shrinkage and iron accumulation and subsequent ROS accumulation, glutathione deficiency, and imbalance in lipid peroxides’ formation and removal. The key regulators of ferroptosis seem to be glutathione peroxidase 4, ferroptosis suppressor protein 1, and oxidized cysteine (cystine)/glutamate antitransporter, known as Xc-system, inhibition of which induces ferroptosis. Ferroptosis has been proposed to play an important role in the occurrence and development of many diseases, including cancer, neurodegenerative disorders, ischemia/reperfusion injury, hematological malignancies, and cardiovascular diseases [65,77,78].

### 3.2. Iron Deficiency

Iron-deficiency mechanisms in patients with HF are not precisely defined. Several mechanisms were postulated to explain the relatively higher prevalence of ID in HF beyond anemia. Moreover, apart from HF, other factors, such as advanced age, kidney failure, female gender, chronic inflammation, malnutrition, reduced iron absorption, and increased iron loss, were independently related to ID in HF patients. Generally, it is assumed that iron deficiency is associated with reduced iron intake/bioavailability, reduced iron absorption, and increased iron loss or combined. However, that seems to be complex, multi-directional, hypothetical, and speculative.

#### 3.2.1. Reduced Iron Intake and Bioavailability

Among HF patients, 35%–58% present malnutrition, especially those with lower protein intake. Malnutrition could be related to several factors, i.e., fatigue, dyspnoea, swallowing disturbances, nausea, anxiety, food monotony, and reduced appetite, additionally related to advice (albeit controversial) to reduce dietary salt intake. Iron bioavailability highly modifies its absorption. Moreover, iron absorption is dependent on the form of iron consumed: heme or non-heme iron. Moreover, other nutrients such as calcium, phytates, and polyphenols (found in tea, coffee, or chocolate) affect iron absorption. Among factors predisposing to ID, inadequate food composition and dietary habits seem to be the main factors, but genetic makeup may also be involved in ID in HF patients.

#### 3.2.2. Reduced Iron Absorption

Right ventricular dysfunction accompanied by venous oedema and congestion is the key element of HF. It reduces intestinal blood supply and causes nonocclusive bowel ischemia, increased mucosal permeability, bowel edema, cachexia, the altered composition of mucosal bacteria, and finally, the malabsorption of micronutrients, including iron.

Moreover, gastric acids provide low pH, which is crucial in converting ferric (Fe3^+^—non-bioavailable) to ferrous (Fe2^+^) iron before absorption by DMT1. So the chronic use of gastric acid-inhibiting medication (routinely prescribed in clinical practice), such as proton-pump inhibitors (PPIs) and histamine-2 receptor antagonists (H2R), increase pH and, thereby, reduce iron absorption.

HF is associated with an increased inflammatory state, following increased levels of several inflammatory markers such as interleukin-1/-6 (IL-1/-6) and tumor necrosis factor alpha (TNF-α). Moreover, the inflammation may be related to hepcidin- and non-hepcidin-related ID mechanisms. Inflammatory markers, mainly IL-6, increase hepcidin expression, which internalizes and degrades the only known iron exporter, ferroportin, thereby leading to a decrease in iron absorption and mobilization from the reticuloendothelial system. It was also shown that other inflammatory markers, e.g., TNF-α, may also lead to impaired iron metabolism in HF, independently of hepcidin [79]. Although the link between this mechanism and ID is potentially possible, no convincing evidence is available.

Moreover, several studies showed that iron deficiency could perpetuate and amplify inflammation. For example, mice with induced ID exhibited enhanced inflammation in post-myocardial infarction (MI). Moreover, in the rat HF model, iron supplementation (FCM) reduced inflammation by dampening the effect of iron on immune cells. Indeed, Toblli et al. [80] showed that administration of iron sucrose intravenously in anemic HF patients led to a significant reduction in C-reactive protein.

#### 3.2.3. Increased Iron Loss

Maintaining iron homeostasis in the body causes a significant reduction in its excretion by stool or urine. In addition, due to the high level of micronutrients in the blood, iron content may be removed from the body during bleeding, including physiological bleeding (menstruation) [21]. Therefore, in HF patients, the prevalence of ID is much more often encountered in the population presented with occult upper and lower GI bleeding, including esophagitis, gastritis, peptic ulcer, inflammatory bowel disease, GI malignancies (gastric, oesophageal, and colorectal cancer), colonic polyps, and angiodysplasia, which are especially frequently found in older patients. Moreover, commonly used cardiology treatments (e.g., antiplatlet, anticoagulant treatment) in vulnerable HF populations with multiple risk factors for GI bleeding, including older age, multimorbidity, and polypharmacy related to intestinal abnormalities, can aggravate occult bleeding.

#### 3.2.4. Iron-Deficiency Consequences

ID’s functional and clinical impairments are observed far beyond mere erythropoiesis. In non-anemic ID, HF patients point toward the important role of iron in nonhematopoietic tissues. The relation between ID and the cellular and subcellular changes is discussed below.

**Mitochondria** are the primary powerhouse of the cell, which utilize glucose, fatty acids, and ketone bodies to produce adenosine triphosphate (ATP). Moreover, they are involved in controlling cellular Ca2^+^, generating reactive oxygen species, cellular death, and synthesizing pyrimidine, amino acids, and lipids [81]. Cardiomyocytes, hepatocytes, nephrons, and skeletal myocytes are the highest energy-demanding cells, hence, they have the highest content of mitochondria. Mitochondria are the major intracellular sites of iron utilization and accumulation. In the utilization of iron, WDR45 beta-propeller scaffold protein plays an essential role. The loss of the WDR45 protein increases cellular iron levels and oxidative stress, accompanied by mitochondrial abnormalities, autophagic defects, and diminished lysosomal function [82]. The proper iron level is crucial for several processes, e.g., synthesis of haeme, iron–sulfur clusters, and mitochondrial biogenesis. Moreover, a reciprocal relationship between adequate iron content and mitochondrial function causes ID to affect iron-containing and non-iron-containing mitochondrial proteins [83,84]. Accordingly, ID leads to morphological changes in the mitochondria (increase in size and decrease in cristae) together with mitochondrial function impairment (reduced production of ATP, biogenesis, impaired mitophagy, mitochondrial DNA damage, increased gluconeogenesis, lactic acid production, and mitochondrial cytochrome c release—hence, apoptosis and reactive species expression), which culminates in mitochondrial damage [85].

**The heart** is especially susceptible to ID, due to having the highest energy expenditure among all organs and being inextricably linked to the mitochondrial function. Myocardial ID induces contractile-function impairment related to reduced activity of respiratory complexes I, II, and III [86]. Systemic ID is associated with worse cardiac function, reduced contractile reserve, and decreased mitochondrial enzymatic activities of oxidative phosphorylation and anti-oxidative enzymes. Similar observations were also made in studies using animal models [87]. Systemic ID is associated with both structural changes: cardiac hypertrophy/fibrosis, irregular sarcomere organization, mitochondrial swelling, and ventricular dilation/hypertrophy; and functional changes: shifts toward anaerobic respiration, impaired mitophagy, oxidative phosphorylation, and heart dysfunction together with lung congestion.

**Skeletal Muscles.** The main symptom in patients with heart failure is exercise intolerance, usually referred to as reduced cardiac output, defective O2 delivery (anemia), impaired skeletal-muscle function, due to anemia-independent pathways related to impaired oxidative metabolism and oxygen storage in the myoglobin [88]. Moreover animal studies have shown that impaired exercise capacity is directly linked to ID due to diminished mitochondrial energy metabolism [84].

**Kidneys.** ID often accompanies renal diseases, however, it is suggested that ID itself affects kidney function. In the nephrons, reabsorption and solute secretion occur as passive or active (e.g., Na^+^/K^+^-ATPase) processes. Therefore, kidneys are high energy-demanding organs with high mitochondrial content. Iron plays a crucial role in the mitochondria, so ID attenuates kidney function, whereas higher iron levels exert a protective effect on renal function [89]. Moreover, it was shown in children that ID was related to increased oxidative stress and tubular glomerular damage [90].

On the other hand, it was proven that IV iron replenishment improves renal function in anemic HF patients with CKD. Similarly, in a subanalysis of the FAIR-HF trial, FCM treatment was accompanied by kidney function (eGFR) improvement, regardless of the presence of anemia [91]. Additionally, ID correction was associated with a reduction in fibroblast growth factor 23 (FGF23), indicating that the beneficial effect of IV iron replenishment is related to an effect beyond the direct effect on the mitochondria [92].

**Other tissues and organs.** Most studies indicate that iron is also essential for brain function, as it is a metabolically active organ susceptible to ID. The energy deficits and ID lead to impaired synaptic plasticity, myelination, and the altered activity of multiple iron-dependent enzymes involved in dopamine and serotonin synthesis. Experimental studies showed that brain ID leads to deficits in memory, learning, behavior, emotional problems, and a relapse of depression (low serotonin level) [93]. Iron homeostasis is multi-directional and related to the immune system. As previously mentioned, inflammation can lead to ID, which affects the immune system in multiple ways. ID negatively influenced both the growth and effector mechanisms of the immune system. The immune system presents several unfavorable effects due to ID, e.g., a defective proliferation of T cells, impaired interleukin 2 production, reduced functions of nuclear factor kappa, HIFs, nitric oxide (NO) formation, and neutrophil activity [94]. ID also impairs thyroid hormone metabolism due to reduced peroxidase activity (heme-containing enzyme), increased hepatic inactivation of thyroid hormones, and ineffective erythropoiesis. Accumulated evidence shows that thyroid dysfunction may be linked to an increased risk for and worsening of HF [95].

## 4. Iron Metabolism in the Failing Human Heart

The proper iron load and its homeostasis are crucial for heart function, as cardiac myocytes require huge amounts of energy produced by iron-containing mitochondrial enzymes. Unfortunately, heart myocytes are also poorly protected from iron overload and sensitive to ROS deleterious effects.

In healthy subjects, iron transport is highly conserved and controlled via several regulatory mechanisms. However, if iron metabolism is perturbed, it may lead to chronic iron-overload-associated morbidity and mortality. The serum transferrin iron-binding capacity is limited, and in case of iron excess, the circulatory iron exceeds transferrin buffering capability, resulting in highly reactive NTBI. Excess uptake of NTBI, combined with the lack of an effective excretory pathway, causes the expansion of the LIP pool, increased ROS formation, and oxidative cellular damage. Cardiomyocytes, like other iron-rich cells, are susceptible to this process. There are no effective pharmacological methods to protect against excess iron import or to facilitate the export of iron from cardiomyocytes in iron overload. In such cases, reduction in iron supplies from the diet (iron-product elimination or chelation therapy) accompanied by enhanced other 2^+^ ions supplementation or bloodletting are the primary treatment methods [96]. As presented above, the possibility of iron-overload occurrence is significantly less frequent than ID in HF, but it should always be remembered, especially in the context of intravenous iron supplementation.

In non-ischaemic, non-advanced HFrEF (NYHA II/III class), Hirsch and coworkers proved that among HF patients, there is a significant variation in the myocardial iron load and no relation with the biomarkers of systemic iron status. However, the lower myocardial iron load is associated with a longer HF history and disease severity—higher serum N-terminal pro-B-type natriuretic peptide levels and also greater LV dysfunction [97]. However, due to the lack of comparison with healthy controls, results should be evaluated with caution.

Generally, most studies conducted in advanced HF proved that the iron load is 15%–32% lower compared to that of non-failing hearts [98]. In chronic heart failure with a reduced ejection fraction (HFrEF), iron deficiency appears in 35%–55% of all patients, depending on the survey [7]. In a meta-analysis of clinical trials in patients with heart failure with preserved ejection fraction, the incidence of iron deficiency is 59% [6]. On the other hand, in patients with acute HFrEF, this number increases to 80% [4]. In HF, both iron deficiency and iron-deficiency anemia are associated with reduced exercise capacity, recurrent hospitalizations, and high mortality due to cardiovascular diseases and deaths from all causes. Therefore, the 2021 guidelines for the treatment of acute and chronic heart failure recommend that all patients with HFrEF should be periodically screened for ID, and if ID is confirmed, intravenous iron supplementation should be considered for the improvement of symptoms, exercise capacity, and the risk of HF hospitalization [99]. The effectiveness of this therapy is based on clinical trials in various groups with HF and iron deficiency—from patients with chronic to acute heart failure. Interestingly, the IRONOUT clinical trial authors reported that oral iron supplementation is ineffective [98]. These suggest that a myocardial iron load did not modify the failing heart function but resulted from a disorder of the proteins involved in the iron turnover. There is, however, little information from human studies to confirm it. Research conducted by Leszek et al. on left ventricle (LV) myocardial biopsies and serum samples revealed a reduced myocardial iron load in a whole group of HF patients, though with a great variety between subjects [100]. Importantly, the myocardial iron level did not affect the advancement of HF [101]. Furthermore, iron content was negatively correlated with myocardial ferritin, which suggests that iron deficiency was associated with disorders with myocardial iron storage. Further studies confirmed that the expression of total myocardial FT and the expression of individual components, FT-HC, FT-LC, and FT-MTC, were significantly reduced in the group of patients with advanced HF [102]. The results suggest more complicated relationships between iron transport and metabolism proteins in the failing heart than previously thought. In the iron-cell-transportation proteins, the expression of TfR1, the crucial transporter for iron uptake, was found to be reduced on the mRNA [42] and protein levels [102]. Moreover, the expression of transferrin-independent iron channels DMT-1 and L-CH were significantly reduced on the protein levels. This study is in line with results obtained in mice with induced by isoproterenol osmotic pump HF, in which both mRNA and protein levels of iron transporters were reduced (DMT-1, TfR-1) [103]. Interestingly, in failing human hearts, there are no changes in the expression of the FPN protein responsible for iron removal from the cell. These suggest that iron deficiency in the heart is additionally related to a higher export and/or a lower import of iron in the cells. These assumptions about reduced DMT-1 and TfR-1 with no changes in FPN concentration were also confirmed by Zhang et al. in the failing human heart [104]. Changes in the expression of iron-metabolism proteins in HF are associated with the modification of iron-regulatory factors. There was a significant reduction in ACO-1 (also known as IREB-1) and IREB-2 proteins in HF patients [102]. Low cytosolic iron levels should acactivate IREBs to increase iron import and decrease iron escape from the cells [105]. This pathway is, however, disturbed in HF patients because low iron levels did not increase IRBs content in the myocardial cells. Moreover, in advanced HF, another transcriptional factor connected to oxygen metabolism HIF-1 is significantly decreased in the cardiac myocytes on the protein [102] and mRNA [106] levels. This led to attenuation of the cardio-protective effect of the HIF system in HF patients.

## 5. Conclusions

The iron myocardial load should be properly balanced, as both deficiency and excess are harmful. The assessment of myocardial iron load is difficult to carry out, and published studies often deliver not-unequivocal results. Future studies should explore the relation among myocardial iron load, HFrEF outcome, and the effects of iron-supplementation therapy. However, iron replenishment should be carried out with caution, together with tight clinical control and analysis of the available parameters characterizing the iron turnover.

## Figures and Tables

**Table 1:** List of the main genes submitted to IRE/IRP post-transcriptional regulation in iron deficiency condition (modified based on [65]). ↑—increase, ↓—decrease.

RNA	Protein	Translation	Effect
FtH/L	Ferritin	↓	↓ Iron-storage protein
DMT1	Divalent Metal Transporter	↑	↑ Iron import
TfR1	Transferrin Receptor	↑	↑ Iron import
Fpn1	Ferroportin 1	↓	↓ Iron export
ACO2	Mitochondrial Aconitase 2	↓	↓ TCA cycle
HIF2α	Hypoxia-Inducible Factor 2α	↓	↓ Hypoxia response

**Table 2:** Iron-overload mechanisms. Modified based on Kowdley et al., 2019 [71].

Classification	Primary Iron Overload	Protein Defect/Mechanism
Type 1A	Homozygosity for the C282Y mutation in HFE—80% of hemochromatosis	Involved in hepcidin synthesis via BMP6, interaction with TFR1
Type 1B	Mutation in HFE gene—H63D, S65C—2%–10% of cases	Involved in hepcidin synthesis via BMP6, interaction with TFR1
Type 2A	HJV mutation	Involved in hepcidin synthesis, BMP co-receptor
Type 2B	HAMP mutation	Downregulation of iron efflux from erythrocytes
Type 3	TFR2 mutation	Involved in hepcidin synthesis, interaction with transferrin
Type 4	SLC40A1 (FPN) mutation	Duodenal iron export
**Classification**	**Secondary Iron Overload**	**Protein Defect/Mechanism**
Iron-loading anemias	Hemoglobin synthesis disturbances—thalassemia major, hemoglobin H The abnormal breakdown of red blood cells—chronic hemolytic anemia, sickle cell anemia, pyruvate kinase deficiency hereditary spherocytosis Red blood cells’ synthesis disturbances— aplastic anemia	Excessive release of iron from red blood cells, chronic transfusions lead to iron overload as humans can not actively remove excess iron,
Parenteral iron overload	RBC transfusions, iron-dextran injections, long-term hemodialysis	Patients requiring recurrent transfusions due to anemia or CKD
Chronic liver disease	Alcoholic liver disease, **non-alcoholic fatty liver disease**, viral liver disease—hepatitis C, hepatitis B, metabolic diseases—porphyria cutanea tarda dysmetabolic iron overload syndrome,	Hepcidin deficiency
Miscellaneous	malignancy (HCC, breast cancer, hematologic malignancies), chronic inflammatory states (systemic lupus erythematosus, rheumatoid arthritis)	Increased iron absorption (possibly from elevated growth differentiation factor 15)
Other	HCC, hepatocellular carcinoma, NAFLD, non-alcoholic fatty liver disease	Hepcidin dysregulation due to insulin resistance

## Data Availability

Not applicable.
